# Mitochondrial Dysfunction and Protein Homeostasis in Aging: Insights from a Premature-Aging Mouse Model

**DOI:** 10.3390/biom14020162

**Published:** 2024-01-30

**Authors:** Jaime M. Ross, Lars Olson, Giuseppe Coppotelli

**Affiliations:** 1 George and Anne Ryan Institute for Neuroscience, University of Rhode Island, Kingston, RI 02881, USA; 2 Department of Biomedical and Pharmaceutical Sciences, College of Pharmacy, University of Rhode Island, Kingston, RI 02881, USA; 3 Department of Neuroscience, Karolinska Institutet, S-17177 Stockholm, Sweden; lars.olson@ki.se

**Keywords:** aging, mitochondrial dysfunction, autophagy, ubiquitin–proteasome system

## Abstract

Mitochondrial dysfunction has been implicated in aging and age-related disorders. Disturbed-protein homeostasis and clearance of damaged proteins have also been linked to aging, as well as to neurodegenerative diseases, cancers, and metabolic disorders. However, since mitochondrial oxidative phosphorylation, ubiquitin–proteasome, and autophagy-lysosome systems are tightly interdependent, it is not understood whether the facets observed in aging are the causes or consequences of one or all of these failed processes. We therefore used prematurely aging mtDNA-mutator mice and normally aging wild-type littermates to elucidate whether mitochondrial dysfunction per se is sufficient to impair cellular protein homeostasis similarly to that which is observed in aging. We found that both mitochondrial dysfunction and normal aging affect the ubiquitin–proteasome system in a tissue-dependent manner, whereas only normal aging markedly impairs the autophagy-lysosome system. Thus, our data show that the proteostasis network control in the prematurely aging mtDNA-mutator mouse differs in certain aspects from that found in normal aging. Taken together, our findings suggest that severe mitochondrial dysfunction drives an aging phenotype associated with the impairment of certain components of the protein homeostasis machinery, while others, such as the autophagy-lysosome system, are not affected or only minimally affected. Taken together, this shows that aging is a multifactorial process resulting from alterations of several integrated biological processes; thus, manipulating one process at the time might not be sufficient to fully recapitulate all changes associated with normal aging.

## 1. Introduction

Aging is characterized by a decrease of many cellular functions which results in decreased adaptability to stressful stimuli and an increased probability of developing diseases, such as cancer, stroke, and various neurodegenerative diseases. Despite recent advances in molecular biology, the mechanisms underlying aging are still not well-understood [1,2]. The process of aging has been viewed as the result of a stochastic and progressive decline of cellular functions due to the accumulation of instances of damage throughout life, with little or no possibility for effective counteractive interventions [3]. However, a correlation between food intake, growth, and longevity, together with the discoveries of genetic mutations that may affect lifespan, has changed the perspective, such that aging can now be seen as the integrated result of genetic and environmental factors, both of which could possibly be targeted to slow down and/or ameliorate the overall process [4,5,6]. Since the initial report that a single mutation in the daf-2 gene doubled the lifespan of *C. elegans* [5], several additional genes and pathways have been found to affect the aging process [7,8], revealing a role of proteins involved in maintaining cellular protein homeostasis and energy levels. Indeed, aging is frequently characterized by the accumulation of altered proteins and dysfunctional mitochondria, which are two hallmarks of the aging process [9,10].

Cellular protein homeostasis is maintained through the interaction between molecular chaperones that assist in protein folding, and protein degradation occurring mainly via the ubiquitin–proteasome system (UPS) and the autophagy-lysosome system (ALS), which together ensure the removal of unwanted or damaged proteins [11,12,13]. This integrated system is defined as the “proteostasis network” [10,14]. Proteasome-mediated protein degradation differs from lysosomal-mediated proteolysis by operating at neutral pH, mainly degrading short-lived proteins, taking place in a protein complex, and not involving intracellular compartmentalization. Proteasome activity generates small peptides that are further digested into amino acids by the abundant cytosolic endopeptidases and aminopeptidases, while lysosomal degradation directly produces single amino acids [15]. During life, molecular stressors, mutations, and translational errors challenge the proteostasis network. Normally, altered proteins may become refolded by chaperones, and if this is not possible, eliminated via UPS or ALS. During aging, one finds increased protein damage and decreased degradation ability of the UPS and ALS, a result which compromises protein homeostasis and leads to accumulation of defective proteins. This, in turn, may lead to neurodegenerative diseases, cancers, and metabolic disorders [16].

Mitochondria are responsible for many important cellular processes, such as being the main source of ATP via oxidative phosphorylation (OXPHOS), cooperating with the endoplasmic reticulum in buffering calcium, regulating apoptosis, participating in steroid synthesis, and producing reactive oxygen species (ROS). Dysfunctional mitochondria and impaired OXPHOS have been found in aged tissues and in age-associated neurodegenerative diseases, such as Parkinson’s disease and Alzheimer’s disease, and thus a role for mitochondria in these conditions has been suggested [17,18]. Direct evidence supporting a primary role of mitochondria in aging has come from a “knock-in” mouse model (the mtDNA-mutator mouse) that expresses a proofreading-deficient version of the catalytic subunit (PolgA) of the mitochondrial DNA (mtDNA) polymerase, which results in increased mtDNA mutations and/or deletions and leads to mitochondrial dysfunction and a premature aging phenotype [19]. Furthermore, we recently showed that starting life with maternally inherited germline mtDNA mutations is sufficient to aggravate aging phenotypes in both mtDNA-mutator mice and mice with a wild-type nuclear background [20,21].

The functions of mitochondria, UPS, and ALS are tightly interdependent, and it remains to be clarified whether mitochondrial dysfunction per se is sufficient to induce the cellular protein homeostasis perturbations observed in aged tissues. To address this question, we compared mtDNA-mutator mice and aged wild-type mice with respect to UPS and ALS alterations.

## 2. Materials and Methods

### 2.1. Reagents

We used mouse monoclonal anti-beta actin (#ab8226) (1:10,000), rabbit monoclonal anti-proteasome subunit beta type 2 (#ab137108) (1:1000), rabbit polyclonal anti-proteasome subunit beta type 5 (#ab3330) (1:1000), rabbit polyclonal anti-SQSTM1/p62 (#ab91526) (1:1000) (Abcam, Cambridge, UK), rabbit polyclonal anti-proteasome subunit beta type 1 (#sc-67345) (1:1000) (Santa Cruz Biotechnology, Inc., Dallas, TX, USA), polyclonal rabbit anti-ubiquitin (#Z0458) (1:1000) (Dako, Glostrup, Denmark), polyclonal rabbit anti-LC3B (#2775) (WB 1:1000, IF 1:200) (Cell Signaling Technology, Danvers, MA, USA), and polyclonal rabbit anti-LAMP1 (#NB120-19294) (1:1000) (Novus Biologicals, Centennial, CO, USA). Horseradish peroxidase (HRP) conjugate of goat anti-rabbit IgG (#G-21234) (1:5000), HRP conjugate of goat anti-mouse IgG and IgM (#A-10677) (1:5000), and donkey anti-rabbit IgG (#A-31572) (1:1000), (Life Technologies, Carlsbad, CA, USA) were also used. To assess proteasomal and lysosomal proteolytic activities, we used Boc-Leu-Arg-Arg-AMC (#BML-BW8515-0005), Suc-Leu-Leu-Val-Tyr-AMC (#BML-P802-0005), Z-Leu-Leu-Glu-AMC (#BML-ZW9345-0005), and z-Phe-Arg-AMC (#260-131-M005) (Enzo Life Sciences, Farmingdale, NY, USA). Phosphate-buffered saline solution (PBS), Iscove’s modified Dulbecco’s medium (IMDM) supplemented with L-glutamine, fetal bovine serum (FBS), heat inactivated trypsin-EDTA 0.25% solution, penicillin, and streptomycin (Life Technologies) were used for the in vitro studies.

### 2.2. Animals

Homozygous mtDNA-mutator mice and control animals with wild-type nuclear DNA were obtained by crossing mice heterozygous for the mtDNA-mutator allele [19]. All WT mice have a wild-type nuclear genome. However, as we have previously demonstrated, continuous crossings of heterozygous mice will transmit a certain load of germline mtDNA mutations from the mother via the egg cell. Because the mothers used for generating homozygous mtDNA-mutator mice are heterozygous, this mtDNA mutation load is modest, but it does confer a modest degree of resultant earlier signs of aging [20]. Of note, all animals in the current study have the same amount of maternally inherited germline mtDNA mutations, and hence the two conditions of being born with mutations of both PolgA alleles versus being old remains valid when comparing the three types of mice (i.e., severe and increasing somatic mtDNA mutation load, as in PolgA^mut/mut^, versus PolgA^WT/WT^ of the same age, and PolgA^WT/WT^ mice that are much older). All mice were group-housed (<4 animals per cage) based on gender in enriched environments, had access to food (R34; Lactamin/Lantmännen, Stockholm, Sweden) and water ad libitum, and were kept on a 12:12 h light:dark cycle at 22–23 °C. Adequate measures were taken to minimize pain and discomfort. Experiments were approved by the Animal Ethics Committee of the Northern Stockholm region and conducted in accordance with international animal welfare standards.

### 2.3. Tissue Preparation

For preparation of tissue samples, animals were sacrificed by cervical dislocation and the liver and brain were dissected, rapidly frozen on dry ice, and stored at −80 °C.

### 2.4. Proteasome Activity Assay

The three proteolytic activities of the 20S proteasome were determined in liver and cerebellum lysates from Mut, WT, and old-WT mice using commercially available fluorescence probes. Either 25 mg of liver or one-half of the cerebellum were lysed in 1 mL lysis buffer containing 50 mM HEPES (pH 7.5), 5 mM EDTA, 150 mM NaCl, 1% Triton X-100, 2 mM ATP, and 1 mM dithiothreitol as antioxidant. Lysates were centrifuged for 10 min at 12,000× *g* at 4 °C, and the supernatants collected. Protein concentration was measured using a protein assay kit (Pierce™ BCA Protein Assay Kit #23227, Pierce Biotechnology, Rockford, IL, USA) according to the manufacturer’s instructions. Following this, 50 µg of protein was incubated with 50 µM of the 7-aminomethyl-4-coumarin (AMC)-labeled peptides Suc-Leu-Leu-Val-Tyr-AMC (for chymotrypsin-like activity), Boc-Leu-Arg-Arg-AMC (for trypsin-like activity), or z-Leu-Leu-Glu-AMC (for caspase-like activity) in the absence or presence of 50 µM Z-Leu-Leu-Leu-vinylsulfone proteasome inhibitor (Enzo Life Sciences, Farmingdale, NY, USA). Fluorescence (Em/Ex 460/380) was measured every minute for 1 h at 37 °C using a multidetection microplate reader (POLARstar OPTIMA, BMG LABTECH GmbH, Ortenberg, Germany). The 20S proteasome activities for each sample were calculated as the difference in fluorescence intensity between the samples and the background fluorescence values; this was obtained by incubating the fractions with the proteasome inhibitor. Lysosomal proteolytic activity was also measured fluorometrically by incubating lysates with 50 µM z-Phe-Arg-AMC (Enzo) in 100 mM sodium acetate (pH 5.5), 8 mM cysteine hydrochloride, and 1 mM EDTA, and recording the fluorescence every minute for 1 h at 37 °C.

### 2.5. Western Blot

Western blot was performed as previously described [22]. Briefly, either 25 mg of liver tissue or one-half of the cerebellum were lysed in 1 mL of buffer containing 1% SDS, 50 mM Tris-HCl, pH 7.4, 150 mM NaCl, 1 mM EDTA, 1% Triton X-100, and complete protease inhibitor cocktail (Roche, Mannheim, Germany). Lysates were centrifuged for 10 min at 12,000× *g* and 4 °C, and the supernatants were collected. Protein concentration was measured using a protein assay kit (see above). Lysates were then mixed with 1× lithium dodecyl sulfate (LDS) buffer and 1× reducing agent (Life Technologies) and heated for 10 min at 90 °C. Thirty micrograms of total cell lysate were separated by gel electrophoresis (NUPAGE Novex Bis-Tris precast gels, Life Technologies), using gels of 10%, 12%, or 4–12%, in an MOPS running buffer. Proteins were blotted on 0.2 μm nitrocellulose membranes (AmershamTM HybondTM-ECL, GE Healthcare Bio-Sciences Corp, Piscataway, NJ, USA). After incubation with the indicated primary and secondary antibodies, immunocomplexes were detected by chemiluminescence (PIERCE ECL, Thermo Scientific, Waltham, MA, USA). Densitometric measurements of the bands in the western blot analysis were evaluated using appropriate software (ImageJ 1.46r, National Institutes of Health, Bethesda, MD, USA), original western blots can be found at Figure S2.

### 2.6. Adult Mouse Fibroblast Culture and Chloroquine Treatment

Tail clips from Mut and WT mice were placed in 10 cm Petri dishes, washed three times with PBS, and diced into small pieces with a razor blade. Tissue pieces were collected in 15 mL Falcon tubes and spun down at 1000× *g* for 5 min. After removing the PBS, 1 mL of a 0.25% trypsin-EDTA solution was added to the tissue, followed by 37 °C incubation for 30 min with 350 rpm shaking. After digestion, cells were collected by 1000× *g* centrifugation for 5 min, and the 0.25% trypsin-EDTA solution was replaced by IMDM containing L-glutamine supplemented with 20% FBS, 100 IU/mL penicillin, and 50 μg/mL streptomycin. Cells were plated in a 100 mm Petri dish and grown in a 37 °C, 5% CO_2_ humidified incubator. After reaching 70% confluence, cells were split 1:3 to 1:4 into T75 flasks and the percentage of FBS in the medium was lowered to 10%. For assessing the autophagic flux in primary fibroblasts, cells were used between passage 6 and 10. Briefly, 500 × 10^3^ cells were seeded in a 6-well plate, and on the following day were treated with 50 μM chloroquine (CQ) for 6, 12, or 24 h. Following this, the cells were harvested and lysed in a pH 7.4 lysis buffer containing 1% SDS, 50 mm Tris-HCl, 150 mm NaCl, 1 mm EDTA, 1% Triton X-100, and protease inhibitors. Protein concentration was measured using a protein assay kit (see above). Protein separation and blotting on nitrocellulose membranes were carried out as described above, and the membranes were probed with an anti-LC3B specific antibody (1:1000, Cell Signaling Technology).

### 2.7. GFP-LC3B Puncta

For GFP-LC3B puncta analysis, fibroblasts were grown on round cover slides for 48 h and then transfected with 0.5 μg of GFP-LC3B plasmid [23] (TurboFect Transfection Reagent, Thermo Fisher Scientific, Pittsburgh, PA, USA), according to the manufacturer’s specifications. After 48 h, cells were treated for 6 h with 50 μM chloroquine (CQ) and then fixed with 4% formaldehyde in PBS for 20 min. After 3 cycles of 5-min washes, the cover slips were mounted on glass slides (Vectashield mounting medium, Vector Laboratories Inc., Burlingame, CA, USA) and sealed with clear nail-polish. Digital images were captured by fluorescence microscopy (Zeiss Axio Imager.M2 microscope, Oberkochen, Germany) and the numbers and sizes of GFP-LC3 fluorescent puncta were quantified using appropriate software (ImageJ 1.46r).

### 2.8. Mitochondrial Analysis

For fluorescence analysis of mitochondria, fibroblasts were grown on round cover slips and transfected with a mitochondrial marker plasmid (mitoKaede expressing plasmid #28133, Addgene, Cambridge, MA, USA) [24]. Transfections (TurboFect reagent, Thermo Fisher Scientific, see above) were performed according to the manufacturer’s instructions. Images were captured and analyzed as described above.

### 2.9. Lipofuscin Detection

Frozen brains were embedded (Tissue-Tek, Sakura Finetek, Torrance, CA, USA) and 14 μm cryostat (Microm Model HM 500M Cryostat, Microm, Germany) sections were taken at −21 °C, thawed onto slides (Menzel-Gläser, Braunschweig, Germany), and stored at −20 °C until use. For auto-fluorescence visualization of lipofuscin deposits, slides were air-dried at room temperature for 1 h, rehydrated in PBS for 5 min, post-fixed with −20 °C methanol for 10 min, washed with PBS for 5 min, mounted (Vectashield, see above), and sealed with clear nail-polish. Lipofuscin auto-fluorescence was observed by excitation at 547 nm and DAPI fluorescence was detected at 405 nm. Digital images were captured (Zeiss LSM510 META Confocal microscope, Zeiss, Oberkochen, Germany) and analyzed (ImageJ 1.46r).

### 2.10. Statistical Analysis

Data are shown as mean ± S.E.M., and the numbers of replicated experiments are indicated in the figures. Statistical analysis was performed with an alpha level of 0.05, by using appropriate statistical tests (Prism v.10, GraphPad Software, San Diego, CA, USA), as indicated in the figure legends. Analysis for outliers was performed for each data set using the Iglewicz and Hoaglin robust test for multiple outliers with a Z score of 3.5. In the analysis, *p* ≤ 0.05 (*), *p* ≤ 0.01 (**) and *p* ≤ 0.001 (***) were considered significant.

## 3. Results

### 3.1. Effect of Mitochondrial Dysfunction and Aging on the Ubiquitin–Proteasome System

In order to assess the possible effects of mitochondrial dysfunction on UPS and ALS, focusing on macroautophagy, referred to hereinafter as autophagy, and to compare such effects with what occurs in normal aging, we measured chymotrypsin-, trypsin- and caspase-like activity of the 20S proteasome. Studies have indicated that tissues comprised of long-lived cells are particularly sensitive to accumulation of damaged structures during aging [25]. We thus tested two different tissues: liver, in which cells retain their ability to divide and regenerate; and cerebellum, largely consisting of postmitotic cells. Activity was measured in 45 ± 2-week-old mtDNA-mutator (Mut) mice, 45 ± 2-week-old wild-type (WT) mice, and >120-week-old wild-type (old-WT) mice.

**Figure 1:**
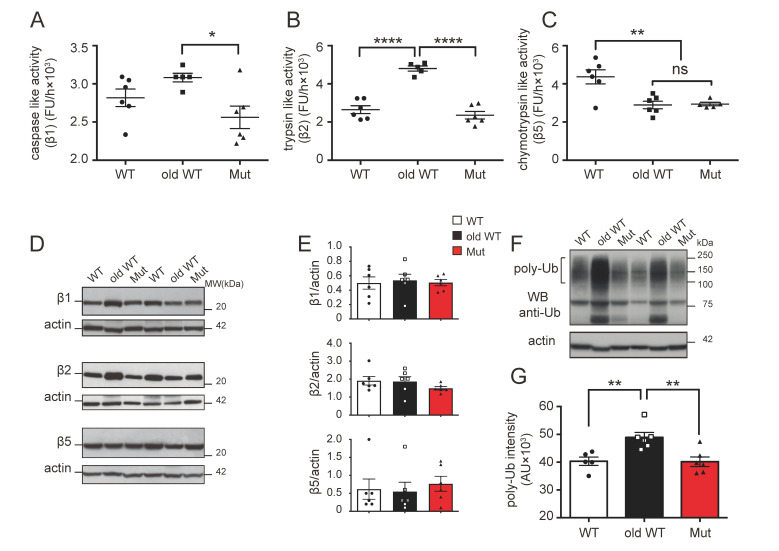
Ubiquitin–proteasome system activity in the liver: (**A**–**C**) caspase- (β1), trypsin- (β2), and chymotrypsin-like (β5) activities of the 20S proteasome in liver lysates from 45 ± 2-week-old wild-type mice (WT), >120-week-old wild-type mice (old-WT), and 45 ± 2-week-old mtDNA-mutator mice (Mut). Mitochondrial dysfunction and aging both significantly affected chymotrypsin-like activity. The activities for WT vs. old-WT vs. Mut were caspase-like: 2820 ± 114 vs. 3080 ± 56 vs. 2560 ± 146 FU/h; trypsin-like: 2650 ± 206 vs. 4800 ± 137 vs. 2350 ± 198 FU/h; and chymotrypsin-like: 4370 ± 372 vs. 2890 ± 197 vs. 2940 ± 91.37 FU/h (one-way ANOVA and Tukey’s post hoc analysis). (**D**,**E**) Level of 20S proteasome catalytic subunits β1 (PSMB1), β2 (PSMB2), and β5 (PSMB5) were assessed by western blot. No differences in protein expression were found between the three groups. One representative western blot and the quantification of the protein levels calculated by normalizing the band intensity with the corresponding actin in six mice are shown. (**F**,**G**) Accumulation of poly-ubiquitinated proteins was assessed by western blot. One representative western blot and the quantification of ubiquitinated protein levels in six mice are shown (one-way ANOVA and Tukey’s post hoc analysis). Data shown are mean ± S.E.M with *p* ≤ 0.05 (*), *p* ≤ 0.01 (**), *p* ≤ 0.0001 (****); ns = non-significant; FU = fluorescence unit, AU = arbitrary unit. Original figures can be found in Supplementary Materials.

We found that mitochondrial dysfunction and aging affected proteasomal caspase-like activity in several ways in the liver. In sum, ~45-week-old mtDNA-mutator mice had lower caspase-like activity, as compared to >120-week-old WT animals, and also a non-significant tendency for a lower caspase activity compared to age-matched WT mice (Figure 1A). Old WT mice also had a slightly higher activity, as compared to the younger WT animals. For trypsin-like activity, we found no difference in mtDNA-mutator mice, as compared to age-matched WT animals, while the activity was significantly higher (≈81%) in old-WT mice, as compared to both the other groups (Figure 1B). Of the three activities, the chymotrypsin-like activity was the only one that was affected in a similar way by both mitochondrial dysfunction and aging, with a ~34% decrease in activity in both prematurely aging mtDNA-mutator mice and old WT mice, as compared to younger WT animals (Figure 1C).

To examine if the differences observed in proteolytic activities may reflect a change in the protein levels of the three proteasomal catalytic subunits (beta 1 (β1, caspase-like), beta 2 (β2, trypsin-like), and beta 5 (β5, chymotrypsin-like)), we next measured levels of these proteins by western blot and found no differences between the animal groups; this suggested that the observed changes in activity were not due to differences in protein abundance (Figure 1D,E). Since the function of the proteasome is to degrade proteins which are targeted for degradation by a covalently linked poly-ubiquitin chain [11], we determined the levels of high-molecular-weight poly-ubiquitinated proteins in the three groups of animals by western blot. Surprisingly, we found that, while dysregulation of proteasome function induces an accumulation of poly-ubiquitinated substrates in the livers of >120-week-old WT mice as compared to ~45-week-old WT mice, no excess accumulation of poly-ubiquitinated protein was present in the lysates of ~45-week-old mtDNA-mutator mice, despite the impairment of proteasome function observed in these mice (Figure 1F,G).

**Figure 2:**
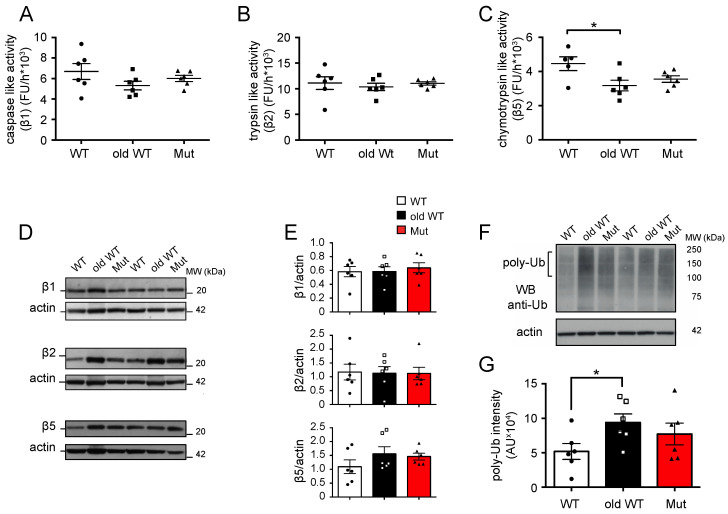
Ubiquitin–proteasome system activity in the cerebellum: (**A**–**C**) caspase- (β1), trypsin- (β2), and chymotrypsin-like (β5) activities of the 20S proteasome in cerebellum lysates from 45 ± 2-week-old wild-type mice (WT), >120-week-old wild-type mice (old-WT), and 45 ± 2-week-old mtDNA-mutator mice (Mut). Chymotrypsin-like activity was significantly decreased in old-WT as compared to WT mice. The activities for WT vs. old-WT vs. Mut were as follows: caspase-like, 6680 ± 773 vs. 5310 ± 419 vs. 6000 ± 305 FU/h; trypsin-like, 11,100 ± 1220 vs. 10,370 ± 720 vs. 11,030 ± 323 FU/h; chymotrypsin like, 4460 ± 401 vs. 3170 ± 311 vs. 3550 ± 196 FU/h (one-way ANOVA and post hoc analysis). (**D**,**E**) Level of 20S proteasome catalytic subunits β1 (PSMB1), β2 (PSMB2), and β5 (PSMB5) were assessed by western blot. No differences in protein expression were found in the three groups. One representative western blot and the quantification of the protein levels calculated by normalizing the band intensity with the corresponding actin in six mice are shown. (**F**,**G**) Accumulation of poly-ubiquitinated protein was assessed by western blot. One representative western blot and the quantification of ubiquitinated protein levels in six mice are shown (Student’s *t*-test). Data are shown as mean ± S.E.M., with *p* ≤ 0.05 (*); FU = fluorescence unit; AU = arbitrary unit. Original figures can be found in Supplementary Materials.

We next repeated the analysis of the three proteolytic activities in the cerebellum, which has a large fraction of postmitotic cells. Unlike the situation in the liver, neither mitochondrial dysfunction nor aging was found to have a major effect on UPS activity in this tissue. There were either no differences or only minor changes in the caspase-like and trypsin-like activities in both the ~45-week-old mtDNA-mutator and >120-week-old WT mice, as compared to the ~45-week-old WT animals (Figure 2A,B). We did find, however, a ~29% decrease in chymotrypsin-like activity in >120-week-old WT mice, as compared to ~45-week-old WT animals (Figure 2C). Neither mitochondrial dysfunction nor aging affected the expression of the three catalytic subunits of the proteasome, namely, β1, β2, and β5 in the cerebellum (Figure 2D,E). Finally, only aging, and not mitochondrial dysfunction, caused significant accumulation of poly-ubiquitinated substrates in the cerebellum, although a tendency for an increase in poly-ubiquitinated proteins was also observed in mtDNA-mutator mice. (Figure 2F,G).

These data demonstrate that mitochondrial dysfunction and aging both affect the UPS in the liver, while no effect or only a minimal effect was found on the UPS in the cerebellum; both conditions induce a decrease in the chymotrypsin-like activity in the liver, while they have an opposite effect on the other two proteolytic activities (Table 1). Interestingly, while such an effect translates into an accumulation of poly-ubiquitinated substrates in liver and cerebellum from older wild-type mice, there is no accumulation of poly-ubiquitinated substrates in mtDNA-mutator mice.

### 3.2. Effect of Mitochondrial Dysfunction and Aging on the Autophagy-Lysosome System

**Figure 3:**
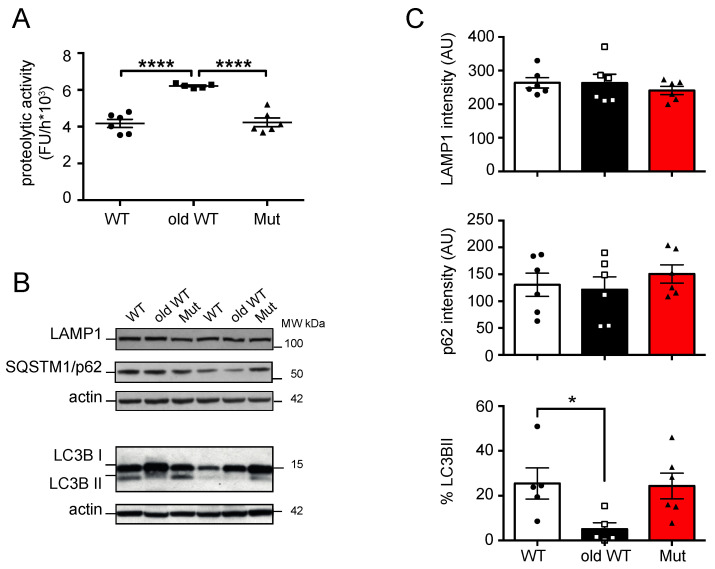
Autophagy-lysosome system activity in the liver: (**A**) cysteine-protease activity in liver extracts from 45 ± 2-week-old wild-type mice (WT), >120-week-old wild-type mice (old-WT), and 45 ± 2-week-old mtDNA-mutator mice (Mut). The activity was significantly increased in liver extracts from old-WT mice as compared to Mut and WT mice. Liver activities for WT vs. old-WT vs. Mut were: 4240 ± 233 vs. 6220 ± 51 vs. 4180 ± 219 FU/h (one-way ANOVA and Tukey’s post hoc analysis). (**B**,**C**) LAMP1, p62 levels, and conversion of LC3B I to LC3B II were assessed by western blot in liver extracts from Mut, WT, and old-WT mice. One representative experiment and the quantification of six mice are shown (one-way ANOVA and Tukey’s post hoc analysis). LC3B II levels expressed as a percentage of total LC3B were 25.5 ± 7, 5.1 ± 2.9 AU, and 24.4 ± 5.7, respectively. Data shown as mean ± S.E.M., with *p* ≤ 0.05 (*), and *p* ≤ 0.0001 (****); FU = fluorescence unit; AU = arbitrary unit. Original figures can be found in Supplementary Materials.

**Figure 4:**
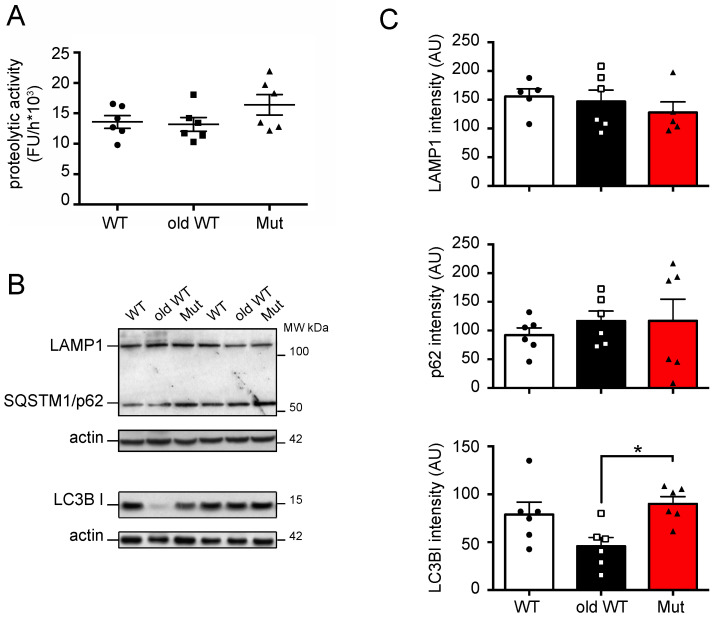
Autophagy-lysosome system activity in cerebellum: (**A**) cysteine-protease activity in cerebellum extracts from 45 ± 2-week-old wild-type mice (WT), >120-week-old wild-type mice (old-WT), and 45 ± 2-week-old mtDNA-mutator mice (Mut). Activities were: 13,600 ± 1050 vs. 13,200 ± 1140 vs. 16,400 ± 1680 FU/h (one-way ANOVA and post-test analysis). (**B**,**C**) LAMP1, p62, and LC3B I were assessed by western blot in cerebellum extracts from WT, old-WT and Mut mice. One representative experiment and a quantification of six mice are shown (one-way ANOVA and Tukey’s post hoc analysis). LC3B I levels in cerebellum were 79.0 ± 12.9, 45.7 ± 9, and 89.9 ± 7.5 AU, respectively. Data shown as mean ± S.E.M., with *p* ≤ 0.05 (*); FU = fluorescence unit; AU = arbitrary unit. Original figures can be found in Supplementary Materials.

Next, we investigated the possible effects of mitochondrial dysfunction and aging on lysosome function and macroautophagy in liver and cerebellum lysates from the three groups of animals. We assessed cysteine protease activity with a fluorogenic substrate (Z-Phe-Arg-AMC) which is cleaved by cysteine proteases, including Cathepsins B, K, L, S, and papain. We found that mitochondrial dysfunction did not affect lysosome function in the liver or cerebellum. Cysteine protease activity was similar in tissues from mtDNA-mutator mice and younger WT mice (Figure 3A and Figure 4A). In contrast, we found a marked 46% increase of cysteine protease activity in the liver tissue from old-WT mice. This result is in agreement with previous reports which suggest this could be a compensatory mechanism attempting to restore lysosomal function [26,27]. No change in the lysosomal marker LAMP1 (lysosomal-associated membrane protein 1) was found by western blot in either tissue type or condition, thus suggesting an absence of change in the lysosomal compartment (Figure 3B,C and Figure 4B,C).

To investigate possible effects of mitochondrial dysfunction and aging on macroautophagy, we next measured levels of SQSTM1/p62 (sequestosome-1) protein, a scaffold/adaptor protein involved in selective macroautophagy, and the conversion of LC3B from the cytoplasmatic form (LC3B I) to the membrane bound form (LC3B II), a commonly used marker for autophagosome formation [28]. We found that SQSTM1/p62 levels were affected neither by mitochondrial dysfunction nor by aging, in both liver and cerebellum from these animals, while aging, but not mitochondrial dysfunction, affected LC3B levels in both tissues (Figure 3B,C and Figure 4B,C). We found an 85% decrease in LC3B II levels in old-WT liver (Figure 3B,C) and a 50% decrease in LC3B I levels in old-WT cerebellum (Figure 4B,C); both are compared to the levels in younger WT mice. Only one species of LC3B protein was detected in cerebellar lysates, which we determined to be LC3B I by comparison with chloroquine-treated fibroblasts that accumulate the LC3B II protein (Figure S1).

Since macroautophagy is a dynamic process and to better understand the effect that mitochondrial dysfunction has on autophagic flux, we established three different adult fibroblast primary cell lines from ~45 week-old mtDNA mutator mice and age-matched wild-type littermates.

**Figure 5:**
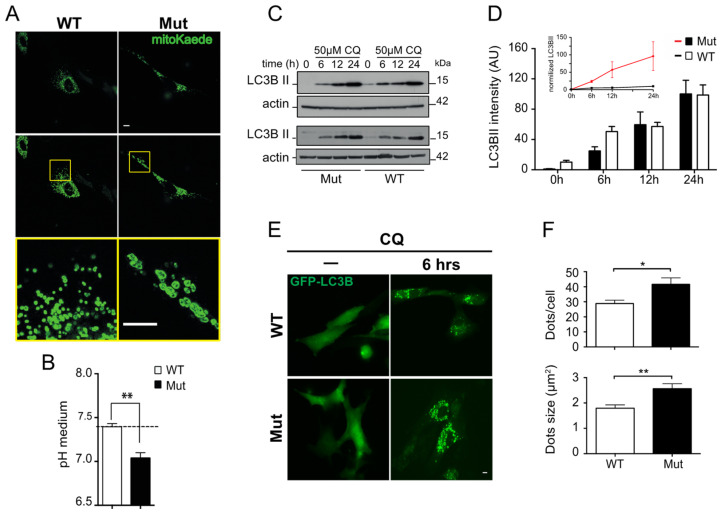
Autophagy-lysosome system in primary-derived cell lines. (**A**) Fluorescence images of mitochondria in fibroblast cell lines derived from WT and Mut animals and transfected with a MitoKaede reporter expressing plasmid. Mitochondria in Mut-derived fibroblasts appear enlarged and swollen, as compared to WT controls. Top panels are the acquired images, while central and bottom images have been enhanced with ImageJ in order to better define the mitochondria. (**B**) Levels of pH in media of three primary fibroblast cell lines from WT and Mut animals. Cells were grown to confluence and then incubated for 36 h in a new medium before measuring pH. The medium from Mut-derived cells had a significantly lower pH as compared to medium from WT-derived cells. Data shown are mean ± S.E.M, with *p* < 0.01 (*n* = 3, Student’s *t*-test). Each cell line was tested twice. (**C**) LC3B II in fibroblasts derived from Mut and WT animals and treated for 6, 12, or 24 h with 50 μM chloroquine (CQ). Western blots for two different cell lines are shown. (**D**) Quantification of three experiments performed with three different cell line pairs is shown, as well as a graph with LC3BII normalized by time zero (upper panel). Data shown as mean ± S.E.M. (**E**,**F**) WT- and Mut-derived adult fibroblasts were transfected with GFP-LC3B and treated for 6 h with 50 μM chloroquine. Representative pictures and quantification of GFP-LC3B puncta number per cell and the average size in CQ-treated cells are shown (mean ± S.E.M; *p* < 0.05 (*) and *p* < 0.01 (**); *n* = 30, Student’s *t*-test). Scale bar = 10 μm. Original figures can be found in Supplementary Materials.

The mitochondria appeared enlarged and swollen in fibroblasts derived from mtDNA-mutator mice, as compared to WT, which is a result of the accumulation of mtDNA mutations (Figure 5A). We also noticed that fibroblasts derived from mtDNA-mutator mice relied heavily on glycolysis for ATP production, as shown by strong medium acidification. This finding is most likely due to an increase in lactate production, which we previously showed to be much higher in mtDNA-mutator mice, as compared to wild-type littermates [29]. After 36 h, fully confluent mtDNA-mutator-mouse-derived fibroblasts had an average medium pH of 7.04 ± 0.06 vs. 7.39 ± 0.04 in WT-derived fibroblasts (*p* < 0.01, Student’s *t*-test, Figure 5B). Failure to replace the medium in fully confluent Mut, but not WT fibroblasts, for more than 48 h resulted in plate detachment of the cells followed by cell death. When we blocked the autophagic flux by treating the fibroblasts with 50 μM chloroquine (CQ), we found that LC3B II levels (normalized to time zero) accumulated significantly faster in fibroblasts from mtDNA-mutator mice as compared to WT fibroblasts (Figure 5C,D). Normalizing the basal LC3B II levels can dramatically affect the experimental outcome, and lower LC3B II levels could be due to either an increase in autophagosome clearance or a decrease in macroautophagy initiation. We therefore further investigated macroautophagy in these cells by assessing the number and size of GFP-LC3B positive dots after transfection with a reporter plasmid and treatment for 6 h with chloroquine. We found a significant increases in both the number and size of GFP-LC3B positive dots in fibroblasts from mtDNA-mutator mice treated with CQ, as compared to WT fibroblasts. These findings thus suggest that the strong accumulation of normalized LC3B II found by western blot in chloroquine-treated cells is indeed due to an increase in autophagic flux in mtDNA-mutator-mouse-derived fibroblasts (Figure 5E,F).

### 3.3. Lipofuscin and Mitochondrial Dysfunction

**Figure 6:**
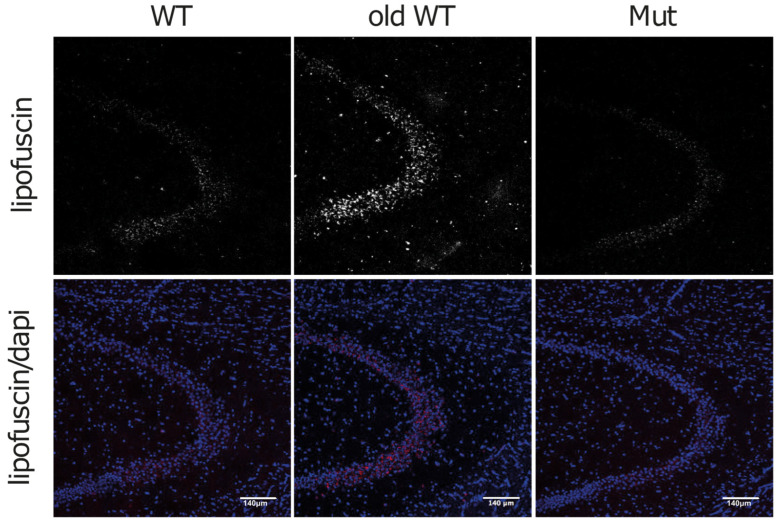
Lipofuscin in the hippocampus of Mut, WT, and old-WT mice. Representative confocal images of the hippocampal CA3 region from 45 ± 2-week-old wild-type mice (WT), >120-week-old wild-type mice (old-WT), and 45 ± 2-week-old mtDNA-mutator mice (Mut), acquired by using an excitation wavelength of 547 nm and an emission filter of 570 nm. Auto-fluorescent lipofuscin granules were clearly visible in old-WT mouse brains. Scale bar = 140 μm.

One hypothesis for the ALS impairment occurring in aging is the progressive accumulation of lipofuscin, the so-called “aging pigment”, in the lysosomal compartment, particularly in muscle and nerve cells [30]. Lipofuscin is a strongly oxidized material, consisting of covalently cross-linked proteins and lipids, mainly derived from the degradation of mitochondria. During normal aging, lipofuscin accumulates in a nearly linear way, thus making it a good marker for aging. Lipofuscin can be detected by its auto-fluorescence using a range of excitation wavelengths, including ultraviolet, blue, and green light [31]. When we analyzed different brain regions from the three groups of animals for auto-fluorescent granules, we found an increase in such granules in the CA3 region of hippocampus of >120-week-old WT brains as compared to ~45-week-old WT brains, but not in the ~45-week-old Mut brains (Figure 6). Taken together, these data support our findings that mitochondrial dysfunction in the mtDNA-mutator mouse have no or minor effects on the autophagy-lysosome system.

## 4. Discussion

A common feature of aging is the accumulation of damaged biological structures due to adverse reactions such as oxidation, glycation, and carbonylation, as well as an overall decline in cellular functions, including UPS, ALS, and OXPHOS [25]. The interconnections of these cellular processes make it difficult to decipher whether a particular event is causing, or the effect of, different aspects of aging. At the cellular level, it is thus difficult to determine if a change is intrinsic to the cell itself or trigged by alterations occurring in other cells and tissues which may result in hormonal and metabolic shifts that affect the rest of the organism [32].

In this study, we used prematurely aging mtDNA-mutator mice and control littermates with a wild-type nuclear genome derived from heterozygous PolgA^wt/mut^ mice to elucidate whether mitochondrial dysfunction per se, caused by being homozygous for the PolgA mutation, is sufficient to impair the UPS and ALS in cells and lead to the generalized perturbation of protein homeostasis typically observed in aging [10,33]. Our data show that the alterations of UPS during aging are complex and differ between tissues (Table 1). These findings are in agreement with previous work [34]. Indeed, we found that, while aging in the liver leads to a generalized dysregulation of the UPS, resulting in accumulation of poly-ubiquitinated proteins, aging in the cerebellum leads to milder or insignificant alterations. Similar results in the cerebellum have been reported in aged rats [35]. We also show that the changes in proteasomal activities during aging in the liver are not unidirectional. While there is a significant decrease in chymotrypsin-like activity, there is an increase in trypsin-like activity, and no change in caspase-like activity. Together, the observed changes may nevertheless result in impairment of proteasome function as indicated by the accumulation of poly-ubiquitinated proteins. Contradictory data are present in the literature on this issue. Our data are in agreement with what was found by Shibatani et al. [36], while other studies report either a more robust decrease or an increase of proteasomal activity [37,38,39]. Such discrepancies could result from differences between the species analyzed, but also from compensatory mechanisms, which could be dependent on genomic background [34].

When we tested the effect of mitochondrial dysfunction on the UPS, we found decreases in two out of the three proteasome-specific proteolytic activities in the liver, while no change was found in the cerebellum (Table 1). Notably, such a decrease in proteasomal activity in mtDNA-mutator-mouse liver was not associated with an increase in poly-ubiquitinated proteins. A decrease in the UPS secondary to mitochondrial dysfunction has been reported in different systems. For example, Huang et al. showed that rat cortical neurons treated with oligomycin, antimycin, or rotenone, which inhibit different elements of the electron transport chain, were found to have reduced 26S proteasome activity. Interestingly, they also found a reduction in E1 activity [40].

The decreased proteasomal activity in liver from mtDNA-mutator mice could perhaps be due to increased oxidation of the proteasomal catalytic subunits. Although initial reports indicated no change in reactive oxygen species (ROS) production in mtDNA-mutator mice [19,41], later studies have actually reported some accumulation of oxidative stress in mtDNA-mutator-mouse tissues [42,43]. Another possibility could be that mitochondrial dysfunction reduces availability of the ATP that is needed for UPS-mediated proteolysis, which could undermine the stability of the proteasome complex and result in disassembly of the lid from the core particle [44]. Moreover, the energy status of the cell could affect post-translational modification of the proteasomal subunits, such as acetylation and phosphorylation [45]. Notably, proteasome impairment might further exacerbate mitochondrial dysfunction in the mtDNA-mutator mouse, since the UPS is an integral component of the mitochondrial quality-control network [46].

It is interesting to compare the effects of normal aging with mitochondrial-dysfunction-driven premature aging with respect to the autophagy-lysosome system. We found that while aging per se can impair the autophagic flux in both liver and cerebellum, which is in accordance with previous reports [38], mitochondrial dysfunction did not have any effect on the autophagic pathway in either tissue, even at 45 weeks of age, when the mtDNA-mutator mouse is near the end of its life. We cannot, however, exclude the possibility that changes in autophagic flux may occur at a younger age in mtDNA-mutator mice and/or in tissues other than those analyzed in this study. In this regard, Li-Harms et al. reported that mitophagy, a selective degradation of mitochondria by autophagy, is impaired in erythroid cells of aged mtDNA-mutator mice [47]; however, this could be due to an impairment in the engulfment of swollen enlarged mitochondria in the autophagosome rather than an effect of autophagic flux reduction [48]. When we analyzed the autophagic flux in primary adult fibroblasts derived from mtDNA-mutator mice, we found an increase in autophagy as compared to fibroblasts derived from wild-type animals. This finding is in line with other studies in different cellular models that report an up-regulation of autophagy in response to mitochondrial dysfunction as one of the compensatory mechanisms for cell survival. For example, mitochondrial dysfunction induced by efavirenz, a reverse transcriptase inhibitor used as an antiretroviral agent, is associated with a compensatory up-regulation of the autophagic system [49]. Moreover, cybrid cells harboring the A8344G mtDNA mutation, the most common mutation responsible for MERRF disease (myoclonus epilepsy with ragged-red fibers), a disorder that affects many organs of the body, particularly the muscular and nervous systems, show up-regulation of autophagy [50]. Our data demonstrate that unlike normal aging, premature aging due to mitochondrial dysfunction is not associated with any significant impairment of ALS, at least not in liver or cerebellum tissue from 45-week-old mtDNA-mutator mice. Moreover, fibroblast cell lines derived from the mtDNA-mutator mouse and harboring mitochondrial dysfunction showed activation of autophagy when compared to wild-type derived fibroblasts.

Our data suggest that the observed mitochondrial dysfunction and impairment of the ubiquitin–proteasome system (UPS) in normal aging could be initiated by a decline in the efficiency of the autophagic machinery. The interconnected nature of these cellular processes suggests a potential cascade effect wherein decreased autophagic efficiency contributes to the accumulation of dysfunctional mitochondria and damaged proteins, subsequently impacting mitochondrial function and UPS activity. In this regard, it has been shown that decreased autophagy, and in particular mitophagy, which is implicated in the removal of damaged mitochondria, induces accumulation of swollen and deficient mitochondria in several models, including the ATG7 knock-out mouse [51], as well as in autophagy-deficient cells, due to the loss of DJ-1 [52]. Accumulation of damaged mitochondria can exacerbate cellular stress and further contribute to the production of ROS, creating a detrimental feedback loop. This, in turn, may intensify the burden on the UPS, as damaged proteins accumulate due to compromised degradation pathways. The consequences of this cellular dyshomeostasis could affect other cellular functions, including energy metabolism, redox balance, and overall proteostasis. Why autophagy activity decreases during aging is still an open question. A possible explanation could be the lysosomal accumulation of lipofuscin, which could impair lysosomal function. Since lipofuscin is composed mainly of mitochondrial proteins and membranes [30], mitochondria might be both primary triggers and effectors of the aging process.

Mitochondria are directly or indirectly involved in a plethora of cellular processes. In addition to ATP production, they regulate apoptosis, calcium signaling, and cellular metabolism, as well as participate in heme and steroid synthesis, cholesterol metabolism, and de novo pyrimidine biosynthesis. It follows that many important cellular mechanisms are disturbed when mitochondria are malfunctioning. Moreover, by regulating the activity of Sirtuins through the NAD+/NADH ratio, mitochondria can also affect cellular gene expression [53], suggesting that mitochondrial dysfunction could perhaps activate a cellular shut-down program, ultimately resulting in aging and death [54].

Taken together, our findings show that the premature-aging phenotype induced by mitochondrial dysfunction does not fully recapitulate the protein homeostasis dysregulation types observed in normal aging. The mtDNA-mutator mouse thus demonstrates that a severe aging phenotype can occur without significant impairment of the autophagic pathway. Additional studies are needed to reveal different mechanisms contributing to aging phenotypes in order to develop effective anti-aging strategies.

## Figures and Tables

**Table 1:** Summary of alterations of proteasomal and lysosomal activity in liver and cerebellum caused by mitochondrial dysfunction and aging.

	PROTEASOME								AUTOPHAGY-LYSOSOME			
	LIVER				CEREBELLUM				LIVER		CEREBELLUM	
Condition	C	T	CT	p-Ub	C	T	CT	p-Ub	PA	LC3B	PA	LC3B
Mitochondrial dysfunction	−	0	−−	0	(−)	0	(−)	(+)	0	(−)	(+)	0
Aging	(+)	+++	−−	+	(−)	0	−	+	+++	−−	0	−

C: caspase-like activity, T: trypsin-like activity, CT: chymotrypsin-like activity, pUb: polyubiquitinated proteins, PA: proteolytic activity, LC3B: microtubule-associated protein light chain 3B. Changes are indicated as: 0 no change, (+) minor increase, + small increase, +++ strong increase; (−) minor decrease, − small decrease, −−medium decrease.

## Data Availability

Original, uncropped, and unadjusted images are available as Supporting Information files. Other data presented in this study are available from the corresponding authors upon reasonable request.

## Supplementary Materials

The following supporting information can be downloaded at: https://www.mdpi.com/article/10.3390/biom14020162/s1, Figure S1: Western blot recognizes LC3BI in cerebellar lysates. Figure S2: Original western blots.
