# Supplementary material for: Mitochondrial Dysfunction and Protein Homeostasis in Aging: Insights from a Premature-Aging Mouse Model
Source: Biomolecules. 2024 Jan 30;14(2):162. doi: 10.3390/biom14020162 (PMC10886786; doi:10.3390/biom14020162)
Supplement: Supplementary file 1 [file biomolecules-14-00162-s001.zip › biomolecules-2776092-supplementary Figure S1.pdf]

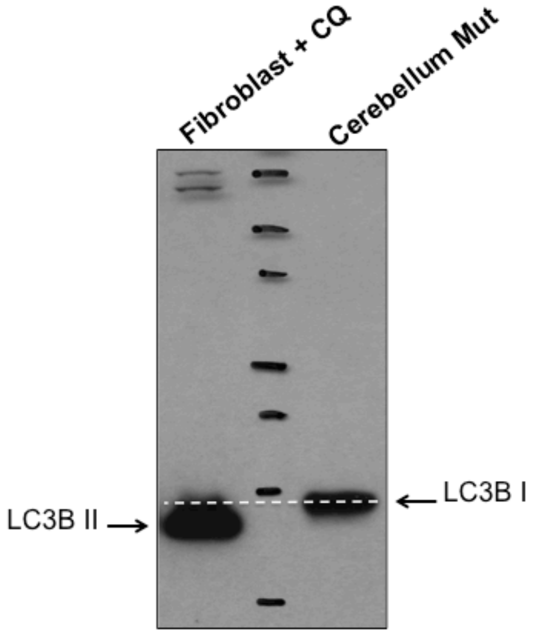

Figure S1. Western blot was used to compare the LC3B II accumulation in chloroquine-treated fibroblast lysates with cerebellar lysates from mtDNA mutator mouse. The size difference of the two protein bands suggests that the protein present in the cerebellar lysate is LC3B I and not II.
