# Supplementary material for: Mitochondrial Dysfunction and Protein Homeostasis in Aging: Insights from a Premature-Aging Mouse Model
Source: Biomolecules. 2024 Jan 30;14(2):162. doi: 10.3390/biom14020162 (PMC10886786; doi:10.3390/biom14020162)
Supplement: Supplementary file 1 [file biomolecules-14-00162-s001.zip › biomolecules-2776092-supplementary Figure S2.pdf]

Figure S2: Original western blots.

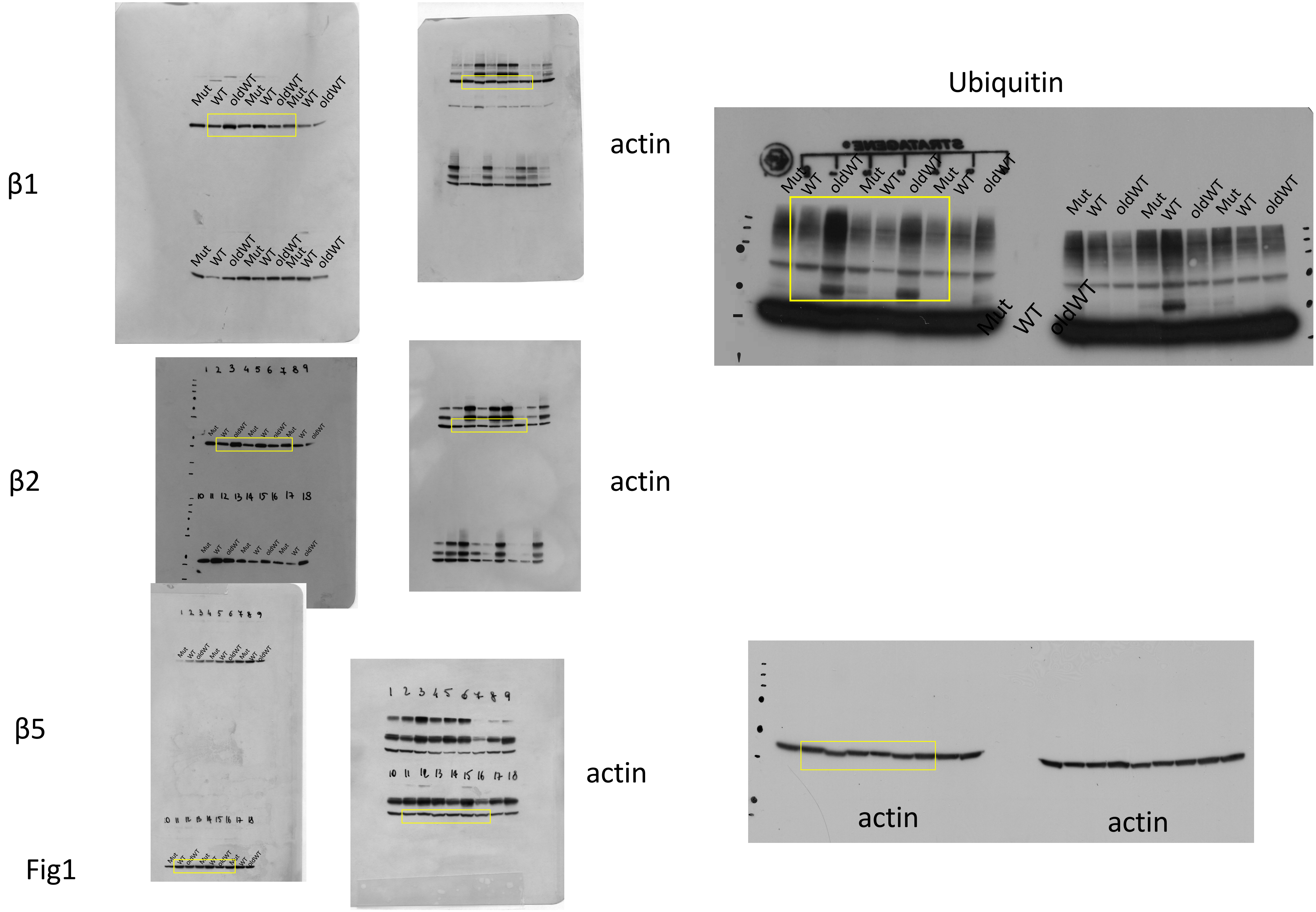

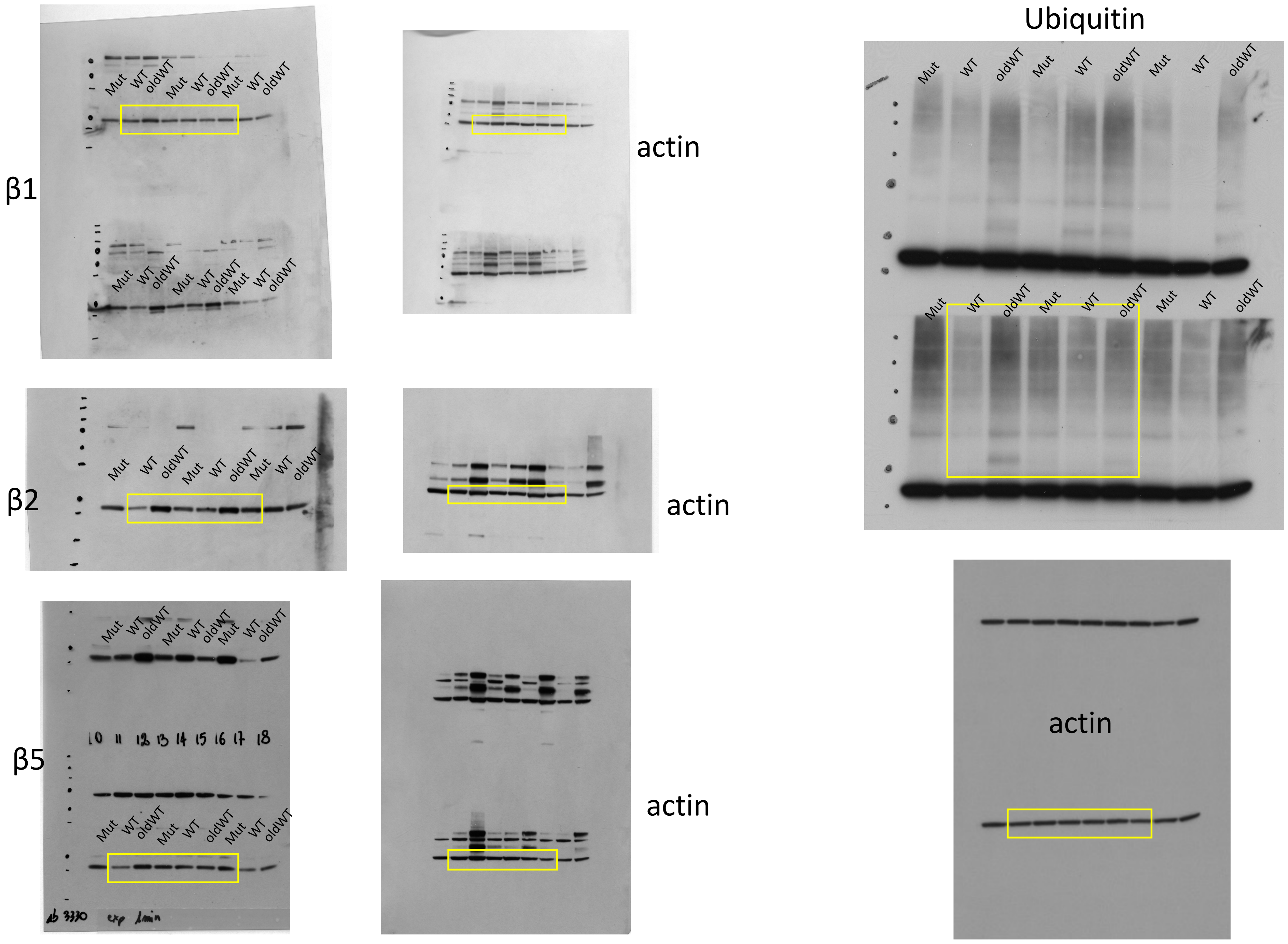
Ubiquitin
β1
actin
β2
actin
β5
actin
actin
Mut WT oldWT Mut WT oldWT Mut WT oldWT
10 11 12 13 14 15 16 17 18
ab 3330 exp 1min

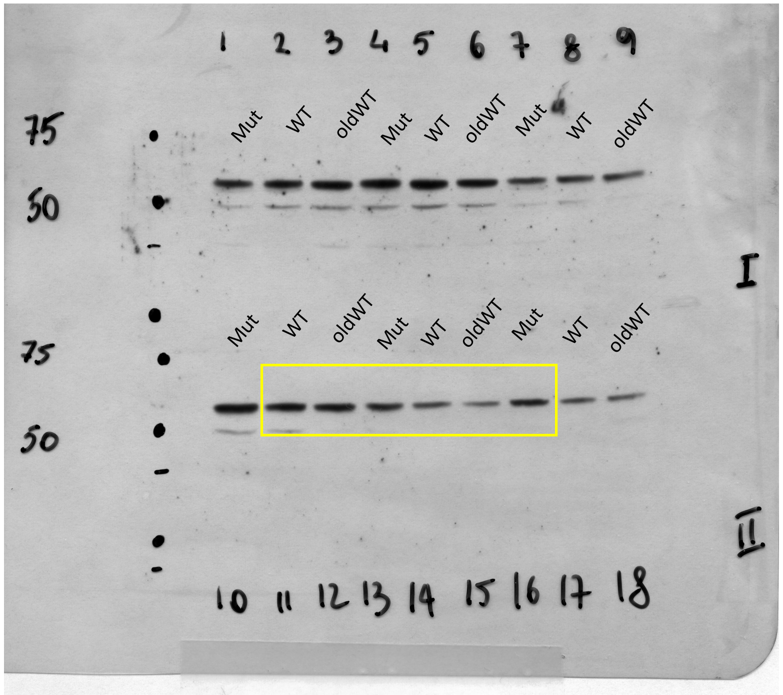

p62

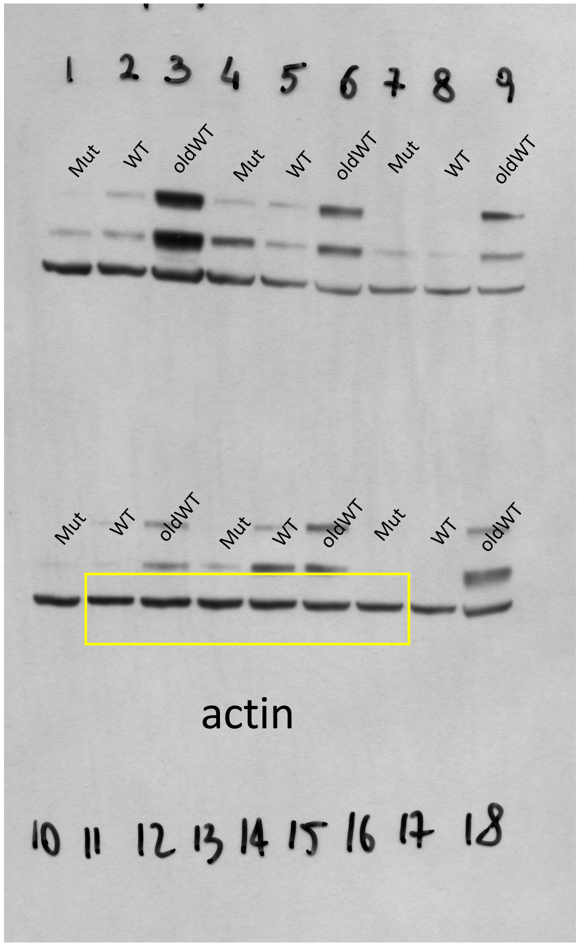

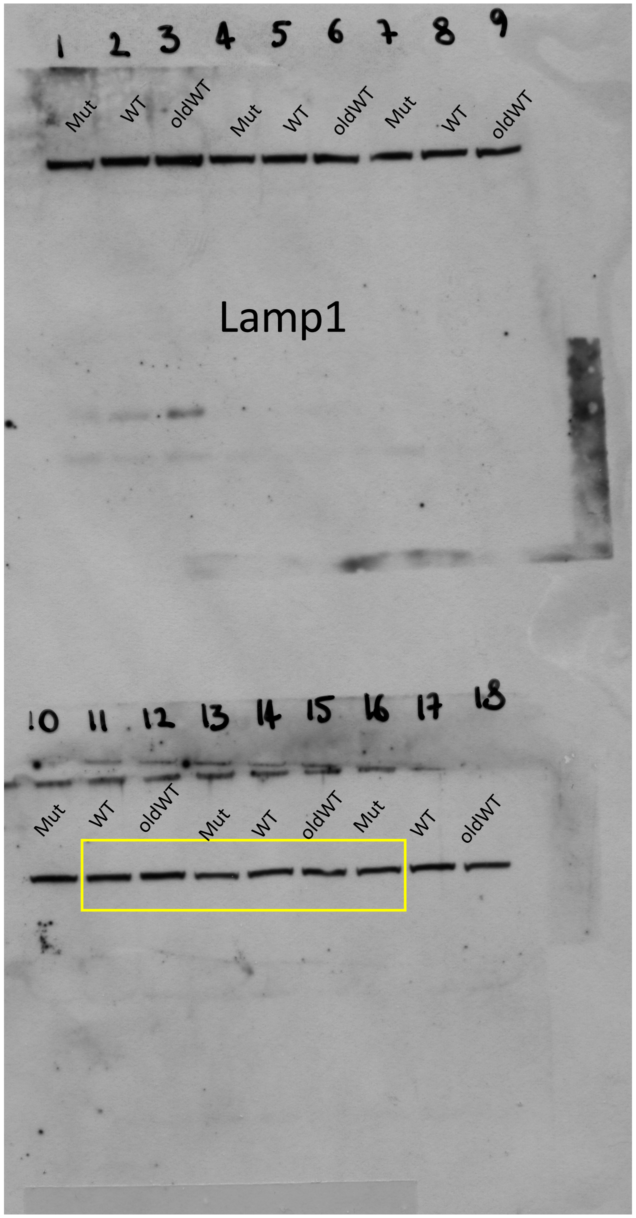

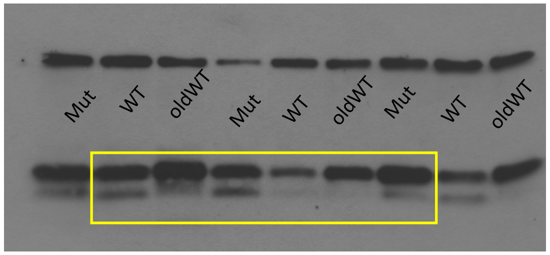

LC3B

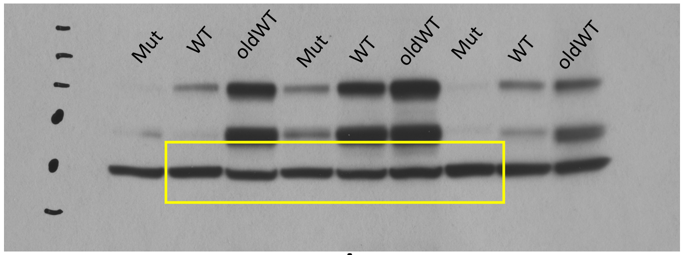

actin

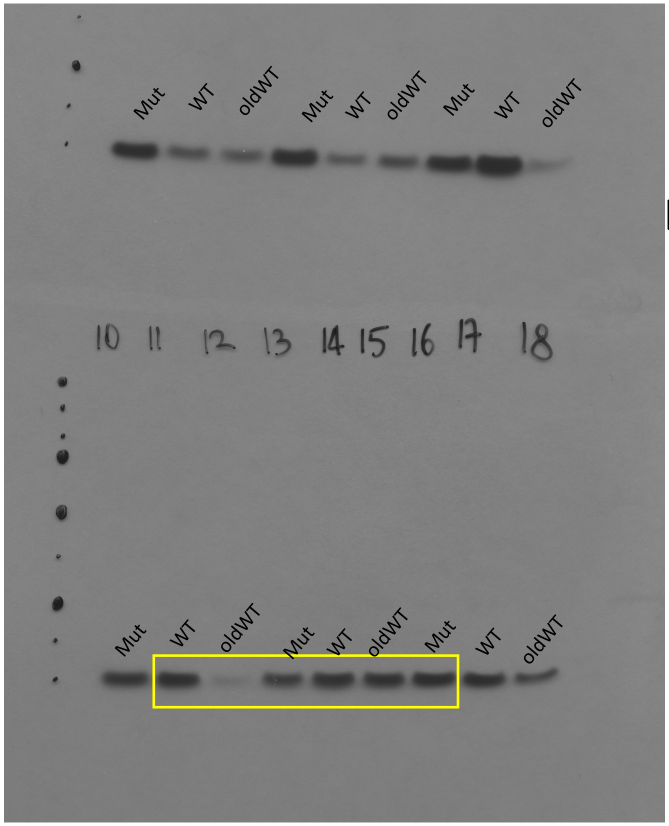

LC3B

Lamp1

p62

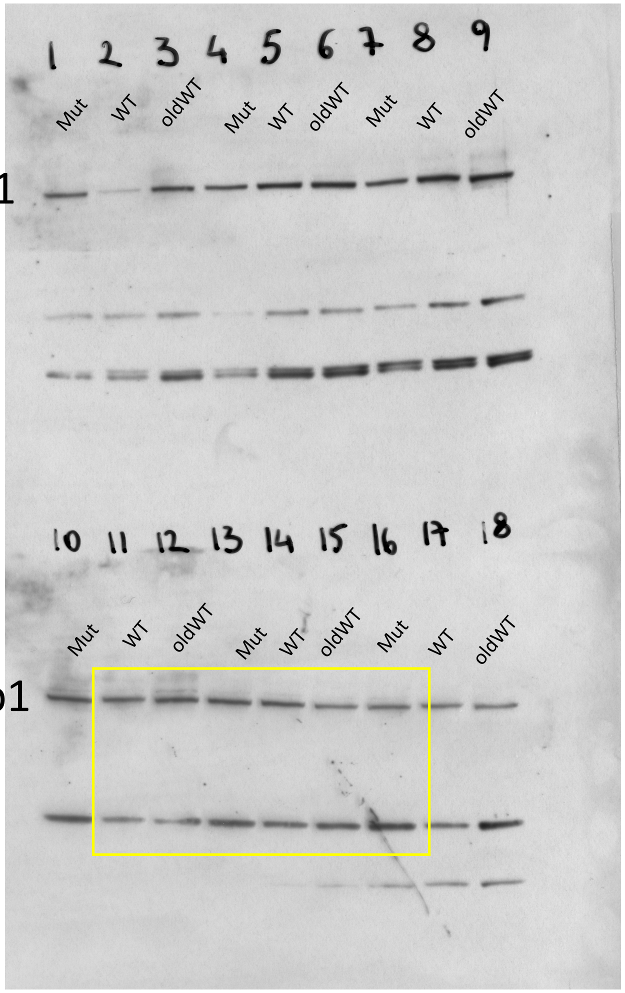

Lamp1

p62

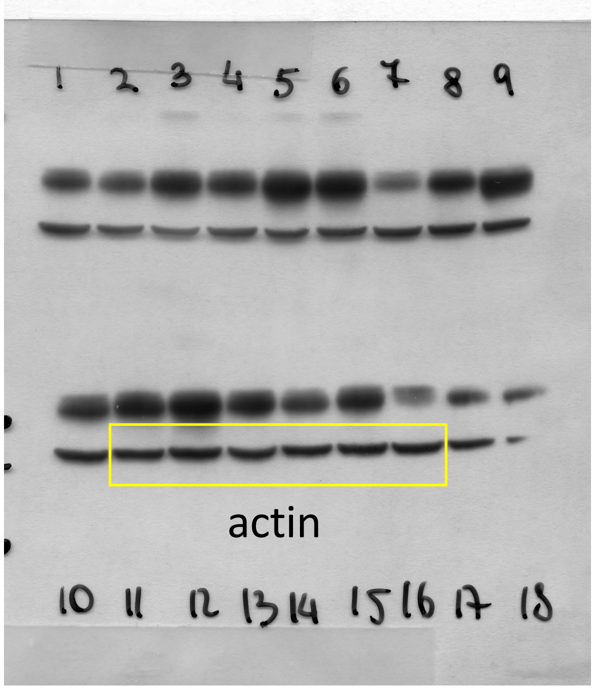

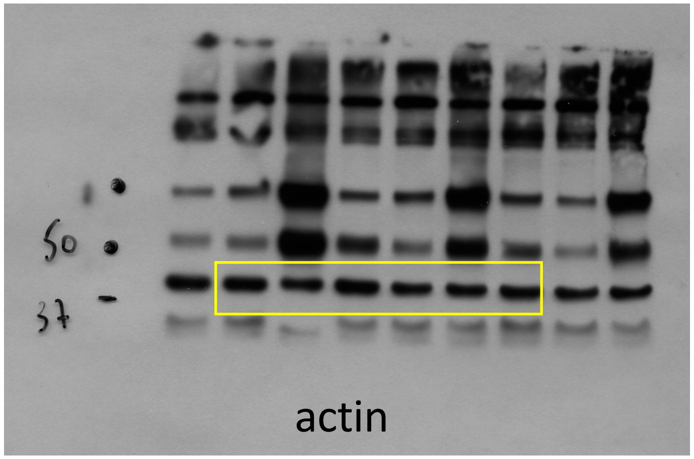

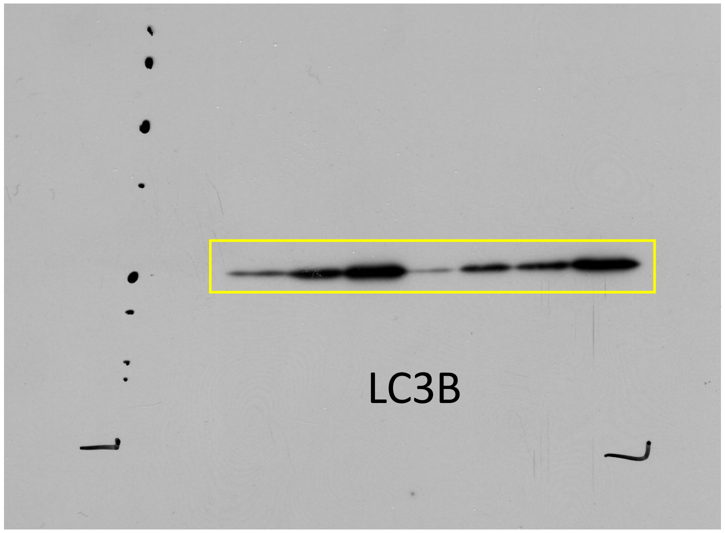
LC3B

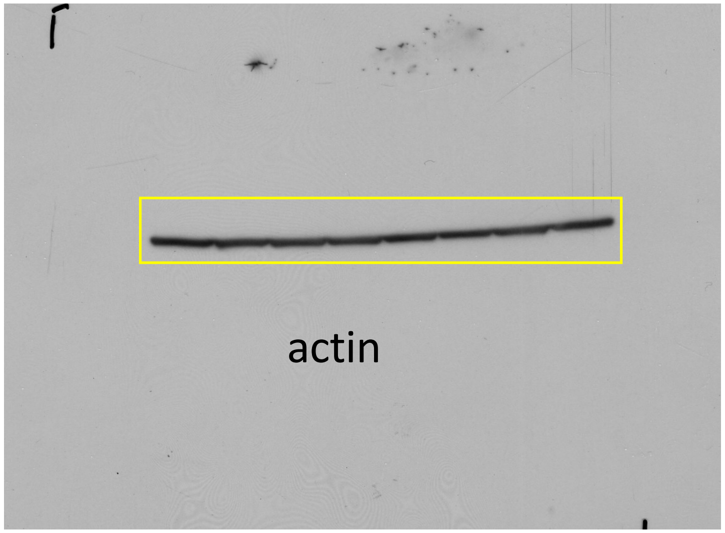
actin

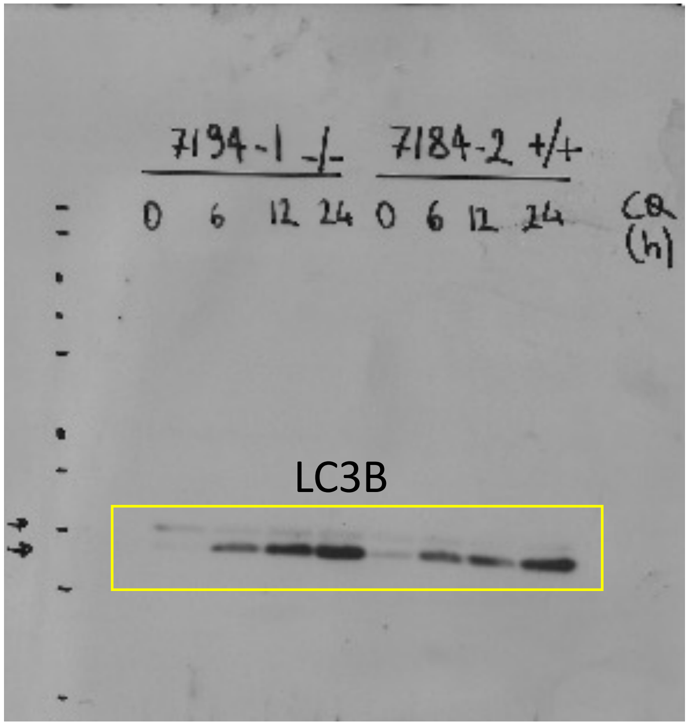
7194-1 -/-
7184-2 +/+
0 6 12 24 0 6 12 24
CQ (h)
LC3B

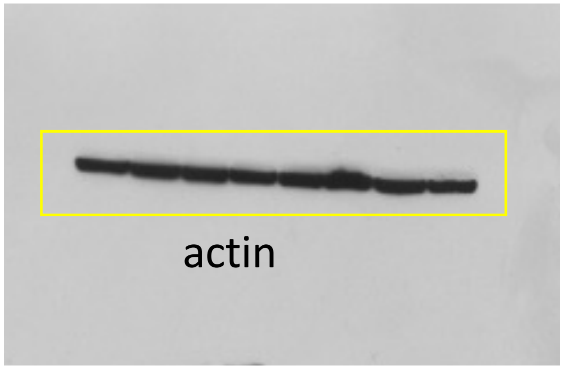
actin
